# High-Fat Diet Induced Gut Microbiota Alterations Associating With Ghrelin/Jak2/Stat3 Up-Regulation to Promote Benign Prostatic Hyperplasia Development

**DOI:** 10.3389/fcell.2021.615928

**Published:** 2021-06-24

**Authors:** Meng Gu, Chong Liu, TianYe Yang, Ming Zhan, Zhikang Cai, Yanbo Chen, Qi Chen, Zhong Wang

**Affiliations:** ^1^Department of Urology, Shanghai Ninth People’s Hospital Affiliated to Shanghai Jiao Tong University School of Medicine, Shanghai, China; ^2^Department of Emergency, Shanghai Ninth People’s Hospital Affiliated to Shanghai Jiao Tong University School of Medicine, Shanghai, China

**Keywords:** benign prostatic hyperplasia, gut microbiota, Ghrelin, Jak2/Stat3, metabolic syndrome

## Abstract

The role of high-fat diet (HFD) induced gut microbiota alteration and Ghrelin as well as their correlation in benign prostatic hyperplasia (BPH) were explored in our study. The gut microbiota was analyzed by 16s rRNA sequencing. Ghrelin levels in serum, along with Ghrelin and Ghrelin receptor in prostate tissue of mice and patients with BPH were measured. The effect of Ghrelin on cell proliferation, apoptosis, and induction of BPH in mice was explored. Our results indicated that BPH mice have the highest ratio of Firmicutes and Bacteroidetes induced by HFD, as well as Ghrelin level in serum and prostate tissue was significantly increased compared with control. Elevated Ghrelin content in the serum and prostate tissue of BPH patients was also observed. Ghrelin promotes cell proliferation while inhibiting cell apoptosis of prostate cells. The effect of Ghrelin on enlargement of the prostate was found almost equivalent to that of testosterone propionate (TP) which may be attenuated by Ghrelin receptor antagonist YIL-781. Ghrelin could up-regulate Jak2/pJak2/Stat3/pStat3 expression *in vitro* and *in vivo*. Our results suggested that Gut microbiota may associate with Ghrelin which plays an important role in activation of Jak2/Stat3 in BPH development. Gut microbiota and Ghrelin might be pathogenic factors for BPH and could be used as a target for mediation.

## Introduction

Benign prostatic hypertrophy (BPH) is characterized by a non-malignant adenomatous overgrowth of the periurethral prostate gland (Woodard et al., 2016). BPH is uncontroversial the most common benign disease of mankind and the prostate inexorably growth often causes troubling symptoms with age (Vuichoud and Loughlin, 2015). BPH is a progressive hyperplasia of prostatic glandular and stromal tissues commonly seen in aging men (Xu H. et al., 2017). Although some risk factors as hormonal, genetic and nutritional factors have been found involved in the etiology of BPH, the pathogenesis of BPH is unclear and more risk factors remain to be further determined (Marker et al., 2003; Gudmundsson et al., 2018).

Metabolic syndrome (MetS) is a progressive condition including a battery of related metabolic abnormalities, such as central obesity, hypertension, hyperglycemia, and insulin resistance (Srikanthan et al., 2016). Central obesity and insulin resistance in particular are recognized as causative factors of MetS. It is widely reported that MetS contributes to the pathogenesis of BPH significantly. According to a previous study, high-fat diet (HFD) may cause prostate enlargement (Xu et al., 2018). Insulin resistance correlated with BPH in rats (Vikram et al., 2010a, b). Data of several epidemiological studies showed that obesity, type 2 diabetes, hyperinsulinemia, and dyslipidemia increase the risk of BPH (Hammarsten and Hogstedt, 2001; Lepor, 2004) and increase the incidence of MetS and BPH in parallel (De Nunzio et al., 2012). MetS may affect the endocrine system, which may contribute to the BPH development (Ngai et al., 2017; Ryl et al., 2017).

Ghrelin is a gastric hormone and mainly secreted by endocrine cells (P/D1 in humans and X/A-like in rats) locating in the gastric fundus. Moreover, Ghrelin expression has also been reported in prostate (Malendowicz et al., 2009; Lu et al., 2012), kidney (Sun et al., 2015), duodenum (Kasacka et al., 2014), colon (Huang et al., 2016), and other organization tissues (Bukowczan et al., 2015; Ercan et al., 2015). As previously reported, Ghrelin is helpful for regulating physical growth, metabolism as well as energy balance and involved in the progress of MetS (Perry et al., 2016; You et al., 2017) and type 2 diabetes (You et al., 2017). Researchers make hypothesis, as strongly supported by more recent evidence, the modulation of Ghrelin-related molecular pathways may be a potential novel target in the treatment of metabolic derangements in disease characterized by metabolic and nutritional complications (Gortan Cappellari and Barazzoni, 2018). Ghrelin was also found in prostate (Gnanapavan et al., 2002) previously. Recent study showed that absolute ventral prostate weight in peripubertal and middle-aged rats was significantly increased by Ghrelin treatment (Plecas-Solarovic et al., 2012).

Gut microbiota plays an important role in host health and disease. Some previous studies suggest that imbalance of gut microbiota have an impact on the metabolic disorders as obesity (Jing et al., 2016; Xu P. et al., 2017) and diabetes (Miele et al., 2015; Munoz-Garach et al., 2016). Microbiota associates with metabolism that it can extract energy from dietary material which cannot be digested (Flint et al., 2008). It has been confirmed that normal food digestion process might be affected by changes in the microbiota ratio *in vitro* and clinical studies. The gut microbiota is a key factor in shaping the biochemical profile of the diet (Rowland et al., 2018) and could have impact on the development of excessive body weight (Isolauri, 2017). It has been found that altered Firmicutes/Bacteroidetes ratio of gut microbiota in animals promotes increased ghrelin secretion correlated with MetS (Perry et al., 2016).

Based on the previous studies, we take a hypothesis that HFD may be associated with gut microbiota change and MetS to induce BPH. In the present study, we explored the effect of gut microbiota alteration in high-fat diet BPH model mice on the level of Ghrelin, and relationship of Ghrelin and BPH development was further investigated. Prostate tissues of patients were used to evaluate the role of Ghrelin, as well as studies *in vitro* and *in vivo* were performed to investigate the proliferative effect of Ghrelin *in vivo* and the effect on prostate enlargement in a mouse model. The signaling pathway of Ghrelin involved in BPH development was also explored.

## Materials and Methods

### Reagents and Antibodies

Recombinant human Ghrelin was purchased from Bio-Techne China Co., Ltd. (Shanghai, China). Testosterone propionate (TP) was purchased from CEN’s industry group (Hangzhou, China). Antibodies including those against Ghrelin and Ghrelin receptor was purchased from Abcam (Abcam, United Kingdom). Antibody against GAPDH was purchased from Proteintech Group, Inc. (Wuhan, China). Others as Jak2, pJak2, Stat3, pStat3, Bcl-2, and BAX were supplied by Cell Signaling Technology (CST) (United States).

### Animals and Experimental Design

The animal studies were conducted and approved by the Shanghai Jiao Tong University Medical Animal Ethics Committees. Eight weeks old male C57BL/6 mice were supplied by Shanghai Experimental Animal Center of Chinese Academy of Sciences (Shanghai, China). Animals were housed in a controlled environment with temperature: 22 ± 2°C; humidity: 50 ± 10%; 12 h light/dark cycle, and all animals had free access to food and water.

For gut microbiota analysis, mice were randomly divided into four groups with 6 mice for each group. (1) Normal group fed a standard chow diet for 12 weeks and injected with 0.9% normal saline daily for 2 weeks (Normal); (2) Control group fed HFD (45% kcal fat/17% kcal sucrose) for 12 weeks and injected with 0.9% normal saline daily for 2 weeks (Control); (3) High-fat diet BPH model group (Model), fed HFD for 12 weeks and received TP (7.5 mg/kg body weight, s.c.) daily for 2 weeks; (4) High-fat diet BPH mice planted with gut microbiota from normal mice group (Model + N_*GM*_), received gut microbiota by intragastric administration once every 2 weeks and fed with HFD for 12 weeks and received TP (7.5 mg/kg body weight, s.c.) daily for 2 weeks. The gut microbiota was isolated from fecal samples of normal mice and cultured on agar media for 48 h. The gut microbiota from one normal mouse was transplanted to a High-fat diet BPH mouse. Mice body weight was measured daily to adjust the dosage. The feces were collected from mice after the last day and then the mice were sacrificed. The tissues of prostate gland were carefully harvested and weighed and then stored at −80°C for further analysis. The prostatic index (PI) (prostate weight mg/100 g body weight) was analyzed.

To further investigate the role of gut microbiota from HFD mice on normal animal and Ghrelin receptor antagonist (YIL-781, Sigma, United States), mice were randomly divided into six groups with five mice in each group. (1) Normal group fed with a standard chow diet for 12 weeks and simultaneously injected with 0.9% normal saline daily for 2 weeks (Normal); (2) HFD group fed with HFD (45% kcal fat/17% kcal sucrose) for 12 weeks and simultaneously injected with 0.9% normal saline daily for 2 weeks; (3) Normal mice planted with gut microbiota from HFD mice group (Normal + HFD_*GM*_), received gut microbiota by intragastric administration once a week and fed with a standard chow diet for 12 weeks. The gut microbiota was isolated from fecal samples of HFD mice and cultured on agar media for 48 h. The gut microbiota from one HFD mouse was transplanted to a normal mouse. (4) High-fat diet BPH model group (BPH), fed HFD for 12 weeks and simultaneously received TP (7.5 mg/kg body weight, s.c.) daily for 2 weeks; (5) BPH with YIL-781 (BPH + YIL-781), BPH mice were injected with YIL-781, 5 mg/kg body weight, i.p. daily for 1 week.

To explore the effect of ghrelin on BPH, mice were randomly divided into three groups with six mice in each group. (1) Control group which injected with 0.9% normal saline daily for 2 weeks; (2) BPH Model group (Model), after castration, received TP (7.5 mg/kg body weight, s.c.) daily for 2 weeks; (3) Ghrelin group, after castration, received Ghrelin i.p.(0.15 mg/kg body weight) for 1 week and received normal saline for another week. Mice body weight was measured daily to adjust the dosage.

Mice body weight was measured daily to adjust the dosage. Fasting blood glucose was detected and the feces were collected from mice after the last day and then the mice were sacrificed. The tissues of prostate gland were carefully harvested and weighed and then stored at −80°C for further analysis. The prostatic index (PI) (prostate weight mg/100 g body weight) was analyzed.

### 16s rRNA Sequencing

Gut microbiota was analyzed according to previous reports (Bode et al., 2013; Dalby et al., 2017). In brief, genomic DNA of fecal samples was extracted by phenol-chloroform, and then column purification. 16S rRNA gene was PCR-amplified using dual barcoded primers. For swimming assays, the PCR product was recovered by gel cutting using the AxyPrepDNA Gel Recovery Kit (AXYGEN), eluted with Tris–HCl; 2% agarose electrophoretic detection. Based on the preliminary quantitative results of electrophoresis, the PCR products were assayed using the QuantiFluor^TM^-ST Blue Fluorescence System (Promega). For MiSeq library construction, using TruSeq^TM^ DNA Sample Prep Kit to connect the “Y” joint and remove the self-ligating fragment of the linker by a magnetic bead and then enrich the library template by PCR amplification followed by denaturation by sodium hydroxide to produce a single-stranded DNA fragment. After MiSeq library construction, MiSeq sequencing was performed on an Illumina MiSeq instrument. One end of the DNA fragment is complementary to the primer base and fixed on the chip; The other end is randomly complementary to another primer in the vicinity and is also fixed to form a “bridge”; PCR amplification to generate DNA clusters and the DNA amplicon is linearized into a single strand; adding the modified DNA polymerase and dNTP with fluorescent labels, only one base per cycle; Scanning the surface of the reaction plate with a laser to read the nucleotide species polymerized in the first round of each template sequence; Chemically cleave the “fluorescent group” and the “termination group” to restore the 3′ end viscosity and continue to polymerize the second nucleotide; Count the fluorescence signal results collected in each round and obtain the sequence of the template DNA fragment.

### Patients and Samples Collection

Patients were systematically diagnosed with BPH on the basis of their urinary syndrome and the ultrasound analysis (prostate volume over 30 ml) (Montorsi et al., 2011) or by pathological analysis after surgery (Xu H. et al., 2017). Our study was approved by the Medical Ethics Committee of Shanghai Jiao Tong University of Medicine and the written informed consent of all the patients involved in our study were obtained. Blood samples from BPH patients were collected during the patients’ hospital visits. The normal blood samples were obtained from blood donation volunteers. The blood samples were centrifuged to obtain the serum and then frozen at −80°C until analysis. Tissues from BPH patients were collected during surgery. And the normal prostate tissues were from a body donor. The tissues were also frozen at −80°C until analysis. All the samples were collected from June 2016 to June 2018.

### Cells and Cell Culture

Human prostate epithelial cell line (RWPE-1) and human prostate stromal cell line (WPMY-1) were obtained from American Type Culture Collection (ATCC, United States). RWPE-1 was maintained in prostate epithelial cells medium (PEpiCM) (ScienCell) with 1% prostate epithelial cell growth supplement (PEpiCGS) (ScienCell) and WPMY-1 were cultured in high-glucose DMEM (HyClone) supplemented with 10% fetal bovine serum (FBS) (HyClone). All cells were incubated at 37°C in an atmosphere with 5% CO_2_. Before treatment, cells were starved for 12 h. The cell lines were treated with 1 μmol/L of Ghrelin referring to previous reports (Abdanipour et al., 2018; Wang Q. et al., 2018) and 1 μM Jak2/STAT3 inhibitor WP1066 (Sigma, United States).

### Cell Proliferation Assay and Three-Dimensional (3D) Culture

Cell proliferation was assayed using Cell Counting Kit-8 (CCK-8) (Dojindo, Japan) assay according to the manufacturer’s instructions. Briefly, 0.5 × 10^5^ cells per well were seeded in 96-well plates. After being cultured with/without Ghrelin for 1–5 days, cells in each well were added 20 μL CCK-8 solution and further cultured for 2 h at 37°C. The absorbance at 450 nm, which is directly proportional to the number of living cells, was detected using an absorbance microplate reader (Thermo, MuLTiSKAN MK3).

Cells treated with/without Ghrelin and WP1066 in three-dimensional (3D) culture assay was performed to detect cell proliferation as described previously (Mi and Xing, 2015; Ko et al., 2018). In brief, the assay was performed in 96-well plates. 1 × 10^5^ cells per well in 100 μL BD Matrigel (BD Biosciences, United States) and 100 μL cell culture with/without Ghrelin covered on the top of the Matrigel. The photographs of colonies growing in the plates were taken on day 1, 3, 5, and 7.

### Flow Cytometric Analysis of Apoptosis

Apoptosis was performed using Cell Apoptosis Kit with Annexin V-APC and PI (Sungene Biotech, China) by flow cytometry according to the manufacturer’ instruction. After treatment with/without Ghrelin and WP1066 for 48 h, cells were harvested and then washed by ice-cold PBS followed by being double-stained with Annexin V-APC and PI. 1 × 10^5^ cells each sample was acquired by a BD FASAria Cell Sorter flow cytometer and the proportions of labeled cells were analyzed using BD Accuri C6 Software (B.D. Biosciences, United States).

### Immunohistochemistry

Specimens from patients and animals in each group were fixed in 10% formalin and then embedded in paraffin, and 5 μm sections were made. After deparaffinisation and hydration, sections were washed by PBS and then with 3% H_2_O_2_ in methanol for 15 min at room temperature. The non-specific binding sites were blocked with normal goat serum. After blocking, the sections were incubated overnight with primary antibodies of Ghrelin (1:500), Ghrelin receptor (1:200), Jak2 (1:400), and Stat3 (1:300) at 4°C. Then the sections were incubated with the corresponding secondary antibody with 0.05% Triton X-100 for 60 min at room temperature and the 3,3’-diaminobenzidine (DAB) was adjusted for color development. And then the sections were counter-stained using hematoxylin. All stained sections photomicrograph were taken (Nikon, Japan).

### Levels of Ghrelin and Ghrelin Receptor Assay

Levels of Ghrelin in the serum of BPH patients and animals were detected using Elisa kits (A102654 for human samples and A106589 for mice samples) purchased from Shanghai Enzyme-linked Biotechnology Co., Ltd. (Shanghai, China) according to the kit’s instructions of the manufacture. Briefly, 50 ul of standard samples were added to each well, and add 50 ul samples to be tested to the sample well followed by incubation at 37°C for 1 h. After the plates were washed for 4–6 times with washing solution, 50 ul of chromogenic substrates A and B was added to each well, protected from light, and incubated at room temperature for 15 min, then 100 ul of stop solution was added to each well and mixed. Measure the absorbance at 450 nm with a microplate reader within 15 min. The data was calculated in the light of standard curves.

### RNA Isolation and Real-Time Quantitative PCR (RT-PCR)

Total RNA was isolated from tissues of patients or animals using TRIzol (Invitrogen, Carlsbad, CA, United States) according to the manufacturer’s instructions. The same amounts of RNA were added to a reverse transcriptase reaction mix (Thermo Scientific, United States), with oligo-dT as a primer. The resulting templates were subjected to PCR using the corresponding specific primers (Table 1).

**TABLE 1:** Sequence of primers.

Name	Primer Sequence (5′-3′)	Products
GAPDH (Forward)	CCCTTAAGAGGGATGCTGCC	263 bp
GAPDH (Reverse)	ACTGTGCCGTTGAATTTGCC	
Jak2 (Forward)	GGCAGCAGCAGAACCTAC	168 bp
Jak2 (Reverse)	CGCCATCCCAAGACACTC	
STAT3 (Forward)	TGGGTGGAAAAGGACATCAG	160 bp
STAT3 (Reverse)	CGGGGTAGAGGTAGACAAGTGG	

### Western Blot Analysis

The prostate tissue from BPH patients was homogenized in cold RIPA buffer and centrifuged at 12,000 × *g* for 10 min at 4°C. Tissue lysates were then subjected to 10% SDS-PAGE and transferred to PVDF (Millipore, Shanghai, China). After blocking in Tris-buffered saline (TBS) buffer with 5% skim milk for 12 h, the membranes were incubated with antibodies against Ghrelin, Ghrelin receptor, Jak2, pJak2, Stat3 pStat3, and GAPDH at 4°C overnight. Then the membranes were washed and incubated with horseradish peroxidase-conjugated secondary antibody for 1 h at room temperature. Blots were detected using enhanced chemiluminescence reagents (Invitrogen, United States), and quantified by densitometric analysis using Image J software. The results were normalized to GAPDH to correct for loading.

### Statistical Analysis

All data are presented as the mean ± SD. Data analysis was performed using GraphPad Prism 6.0 software. Differences between two groups were analyzed using Student’s *t* test, as among three or more groups using one-way ANOVA with Bonferroni correction. Differences were considered statistically significant when *p* value < 0.05.

## Results

### Gut Microbiota Analysis and Ghrelin Assay in BPH Mice

**FIGURE 1:**
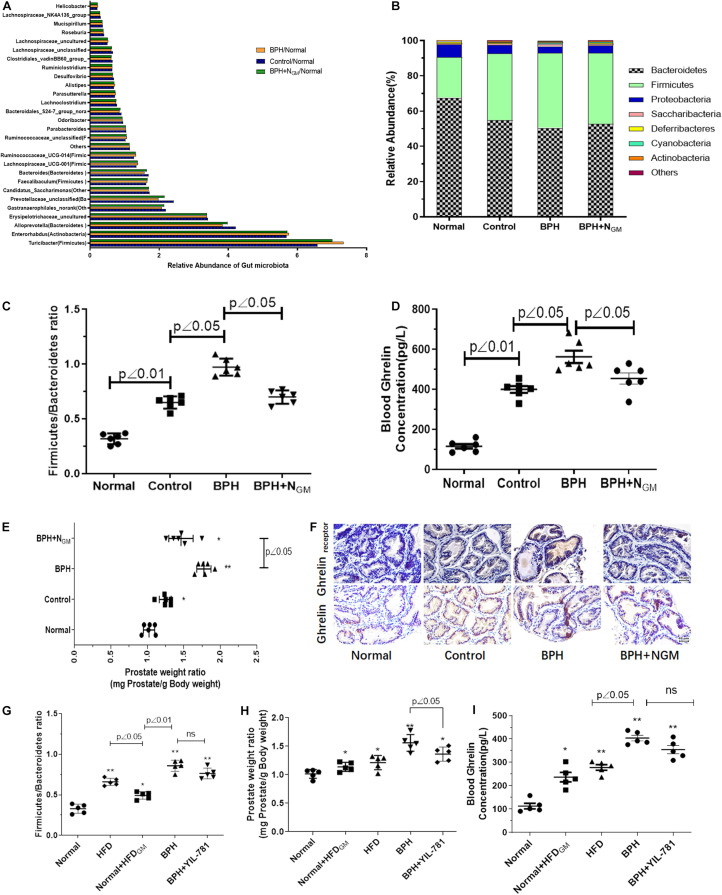
Gut microbiota associated ghrelin to induce BPH in mice. Normal, mice fed with regular chow diet for 12 weeks; Control: mice fed with high-fat diet (HFD) for 12 weeks; BPH group: mice fed with HFD for 12 weeks and received testosterone propionate (TP) (7.5 mg/kg body weight, s.c.) daily for 2 weeks; BPH + NGM: BPH mice planted with gut microbiota from normal mice group. *n* = 6. Gut microbiota was analyzed by 16s rRNA sequencing. **(A,B)** The relative abundance of gut microbiota changes in Normal, Control, and BPH animals. **(C)** The ratio of Firmicutes and Bacteroidetes in Normal, Control and BPH animals was showed. *n* = 6. **(D)** Ghrelin level in serum of animals was measured by Elisa kit. *n* = 6. **(E)** The prostatic index (PI, prostate weight mg/100 g body weight) of mice in Normal, Control, and BPH group. *n* = 6. **(F)** Ghrelin and Ghrelin receptor expression of prostate tissue detected by immunohistochemistry. Normal, mice fed with regular chow diet for 12 weeks; HFD: mice fed with HFD for 12 weeks; BPH group: mice fed with HFD for 12 weeks and received testosterone propionate (TP) (7.5 mg/kg body weight, s.c.) daily for 2 weeks; Normal + HFD_*GM*_: Normal mice planted with gut microbiota from HFD mice group; BPH + YIL-781: BPH mice injected with YIL-781 (5 mg/kg body weight, i.p.) daily for a week. *n* = 5. **(G)** The ratio of Firmicutes and Bacteroidetes in the animals. **(H)** The prostatic index (PI, prostate weight mg/100 g body weight). *n* = 5. **(I)** Ghrelin level in serum of animals was measured by Elisa kit. *n* = 5. *p < 0.05, **p < 0.01 compared with normal.

Gut microbiota was analyzed by 16s rRNA sequencing in our study. As showed in Figures 1A,B, the relative abundance of gut microbiota changes in Normal, Control (HFD fed mice) and BPH animals. The relative abundance of Firmicutes was significantly increased while Bacteroidetes decreased in control (HFD fed mice) and BPH group. It was worth noting that Firmicutes relative abundance was more and Bacteroidetes was even less in BPH group than control. The ratio of Firmicutes to Bacteroidetes in HFD fed mice was significantly increased compared with normal animals. BPH mice have higher ratio of Firmicutes and Bacteroidetes than control animals, which tends to be decreased in BPH mice received gut microbiota from normal animals (Figure 1C). BPH mice induced by TP and fed with HFD tend to have higher blood glucose than normal mice (7.09 ± 1.3 vs. 5.01 ± 0.8).

Ghrelin level in serum of animals was measured by Elisa kit. Compared with normal mice, the serum level of Ghrelin was increased in HFD fed mice (Control and BPH) and BHP mice showed even higher serum level of Ghrelin than control animals and gut microbiota from normal animals induced decreasing level of Ghrelin in serum of BPH mice (Figure 1D). HFD and TP induced prostate of mice enlargement, while gut microbiota from normal animals tend to induce prostate enlargement decreasing (Figure 1E). Results of immunohistochemistry showed in Figure 1F was similar to that of Elisa assay that Ghrelin and Ghrelin receptor expression of prostate tend to be up-regulated in HFD fed mice (Control and BPH) while BPH animals showed relative higher Ghrelin and Ghrelin receptor expression in prostate tissue.

As showed in Figure 1G, gut microbiota from HFD mice tends to induced increasing ratio of Firmicutes to Bacteroidetes of normal group fed with a standard chow diet, which is similar to HFD fed mice. Higher prostate weight ratio and serum level of Ghrelin were also found in normal mice with HFD- gut microbiota compared with normal group (Figures 1H,I). Ghrelin receptor antagonist YIL-781 showed little effect on ratio of Firmicutes to Bacteroidetes and blood ghrelin, however it significantly inhibits the prostate enlargement induced by HFD and TP.

### Expression of Ghrelin and Ghrelin Receptor in Specimens From BPH Patients

**FIGURE 2:**
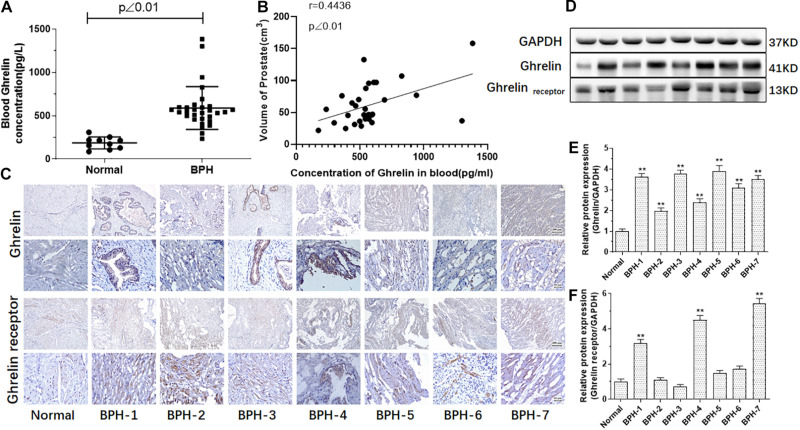
Ghrelin or/and Ghrelin receptor measured in the serum and prostate tissue in the BPH patients. Compared with normal (*n* = 10), Ghrelin levels in the BPH patients (*n* = 30) are significantly increased (*p* < 0.01). **(A)** Ghrelin levels in the BPH patients’ serum might be positively correlated with prostate volume. **(B)** Ghrelin expression analyzed by immunohistochemistry **(C)** and western blotting **(D–F)** was significantly increased in prostate tissue of all BPH patients (*n* = 7) compared with normal prostate tissue while Ghrelin receptor increased in parts of samples of BPH tissue in our study (**p < 0.01 compared with normal prostate tissue).

Total Ghrelin levels of serum samples from 30 BPH patients and 10 normal donors, and protein expression of Ghrelin and Ghrelin receptor (GHSR 1) in seven BPH patients and one normal donor were measured in our study. As showed in Figures 2A,B, Ghrelin levels in BPH patients’ serum were significantly high compared with normal samples (*p* < 0.01) and positively correlated with volume of the prostate (*r* = 0.4736, *p* < 0.01) (Table 2). Body Mass Index (BMI), which is usually considered as standards for obesity, is significantly high in BPH group compared with Normal group, and BPH patients tend to have overweight. Protein expression of Ghrelin in BPH tissues measured by western blotting was higher than that in normal one (Figures 2D–E). For Ghrelin receptor, it’s about 42.9% of samples showed high protein expression significantly compared with normal (Figures 2D,F). Results of western blotting were confirmed by immunohistochemical analysis (Figure 2C).

**TABLE 2:** Clinical data of BPH patients.

Group	Age	BMI (kg/m^2^)	Media PSA (cm^3^)	Media Prostate Volume (cm^3^)
	(Years old)			
Normal	59.5 ± 3.7	22.28 ± 0.6	0.92	21.67
BPH	70.4 ± 5.4	24.32 ± 1.1	2.15	67.28

### Ghrelin Promotes Proliferation and Induces Protein Jak2 and Stat3 Expression of Prostate Epithelial Cells and Prostate Stromal Cells

**FIGURE 3:**
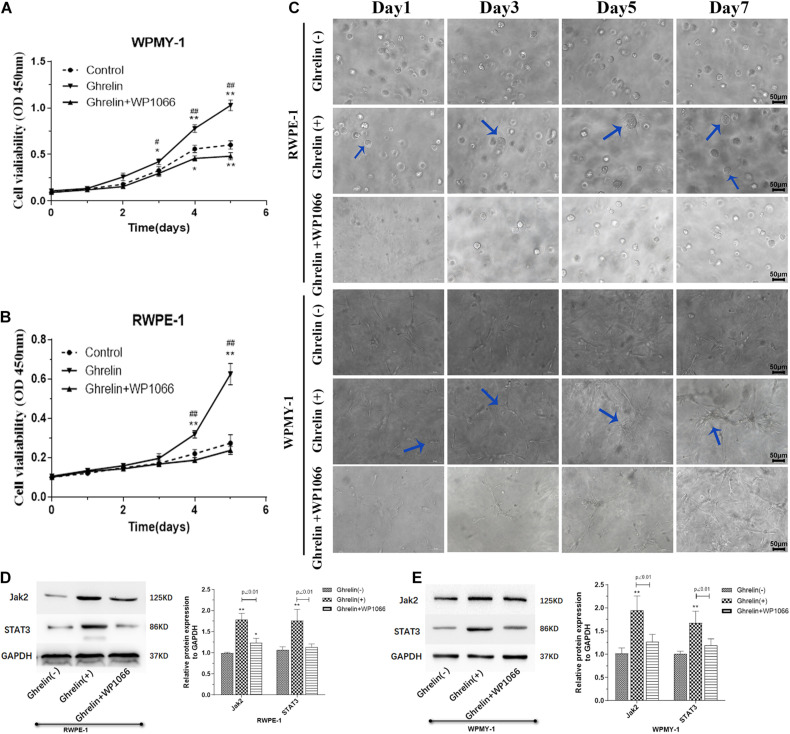
Ghrelin treatment enhanced cell proliferation induces protein Jak2 and Stat3 expression of both prostate epithelial cells (RWPE-1) and stromal cells (WPMY-1). Ghrelin (1 μmol/L) increases the proliferation observed using CCK-8 kit **(A,B)** and 3D cell culture **(C)** which could be attenuated by Jak2/STAT3 inhibitor WP1066 (1 μM). Jak2 and STAT3 expression was significantly up-regulated in Ghrelin treated cells measured by western blotting showed in **(D)** RWPE-1 and **(E)** WPMY-1. (*n* = 5) *p < 0.05, **p < 0.01 compared with Control (Ghrelin(-)); #*p* < 0.05, ##*p* < 0.01 compared with Control (Ghrelin + WP1066).

To evaluate the effect of Ghrelin on BPH *in vitro*, experiments of cell proliferation and apoptosis in human prostate epithelial cells (RWPE-1) and prostate stromal cells (WPMY-1) were performed in our study. It was showed in Figures 3A,B that Ghrelin significantly promoted cell proliferation of both RWPE-1 and WPMY-1 detected by CCK-8, which is inhibited by WP1066, an inhibitor of Jak2/STAT3. 3D culture cells experiment to observe cell proliferation was also performed, results of which indicated that Ghrelin may promote cell proliferation through Jak2/STAT3 pathway, which is consistent with CCK-8 assay (Figure 3C).

The expression of Jak2 and Stat3 in Ghrelin-treated RWPE-1 and WPMY-1 cells was measured by western blotting. Compared with the control group, both Jak2 and Stat3 expression was significantly up-regulated in Ghrelin treated RWPE-1 and WPMY-1 cell lines (*p* < 0.01), which could be inhibited by WP1066 (Figures 3D,E).

### Ghrelin Inhibits Apoptosis of Prostate Epithelial Cells and Prostate Stromal Cells

**FIGURE 4:**
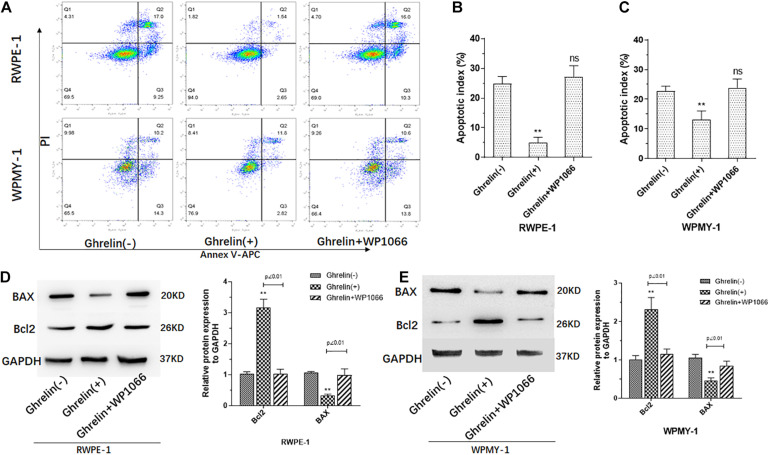
Ghrelin treatment inhibited cell apoptosis of both prostate epithelial cells (RWPE-1) and stromal cells (WPMY-1). Ghrelin (1 μmol/L) reduces the apoptosis of the RWPE and WPMY cell lines **(A–C)** which could be attenuated by Jak2/STAT3 inhibitor WP1066 (1 μM). Bcl2 and BAX expression measured by western blotting confirmed the effect of Ghrelin on apoptosis **(D,E)**. (*n* = 5) **p < 0.01 compared with Control (Ghrelin(-)).

Apoptosis assay of both RWPE-1 and WPMY-1 was also performed to investigate the effect of Ghrelin. As showed in Figures 4A–C, percent of apoptosis cells was significantly low in Ghrelin treated RWPE-1 and WPMY-1, which may be reversed by WP1066. It seemed that Ghrelin showed much more inhibition of apoptosis in RWPE-1 than WPMY-1. Results of protein expression measured by western blotting confirmed the apoptosis assay that Bcl2 expression was increased and BAX decreased in Ghrelin treat cells of both RWPE-1 and WPMY-1 (Figures 4D,E).

### Ghrelin Promotes BPH Progress *in vivo*

**FIGURE 5:**
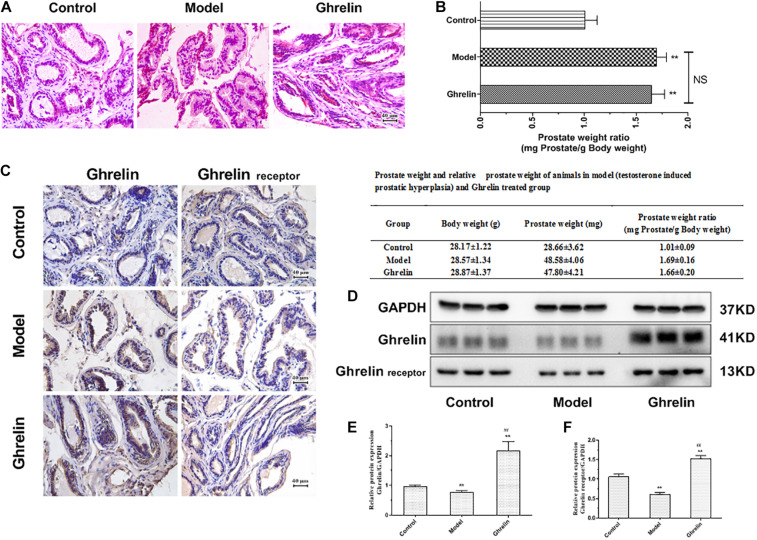
Ghrelin treatment enlarges prostate glands in mice. **(A)** The HE stains of prostatic tissue sections. Increased epithelial cells hyperplasia and enhanced connective tissue with hyperplastic dilated glands were observed in Ghrelin treated mice (after castration, 0.15 mg/kg body weight, i.p., daily for 1 week, *n* = 5). **(B)** The effect of Ghrelin was equivalent to that of Testosterone propionate (TP, 7.5 mg/kg body weight, s.c., daily for 2 weeks, *n* = 5) on the prostatic index (PI) (prostate weight mg/100 g body weight). The expression of Ghrelin and its receptor was increased in Ghrelin treated mice and TP also tended to induce enhanced Ghrelin and Ghrelin receptor expression measured by immunohistochemistry **(C)** and western blotting **(D–F)**. **p < 0.01 compared with Control (*n* = 5, Normal mice with placebo). ##*p* < 0.01 compared with Model (Mice treated with Testosterone propionate, 7.5 mg/kg body weight, s.c., daily for 2 weeks, *n* = 5). The samples for detection in panel **D** are the same as that in Figure 6D and GAPDH blots was the same.

Compared with mice in control group (normal mice with normal saline), animals in model group (BPH) showed increased epithelial cells in the prostate, while both epithelial cells increased and enhanced connective tissue with hyperplastic dilated glands were found in Ghrelin-treated mice (Figure 5A). There was no significant difference found between PI in Ghrelin-treated mice and testosterone-induced (Figure 5B).

In immunohistochemistry study, testosterone tended to induce enhanced Ghrelin and Ghrelin receptor expression (Figure 5C), even though it was not confirmed in western blotting assay. Mice in Ghrelin-treated group showed increased protein expression of both Ghrelin and Ghrelin receptor significantly (*p* < 0.05) compared with control group (Figures 5D–F).

### Ghrelin Up-Regulated Jak2/Stat3 Expression in Prostate of BPH Mice

**FIGURE 6:**
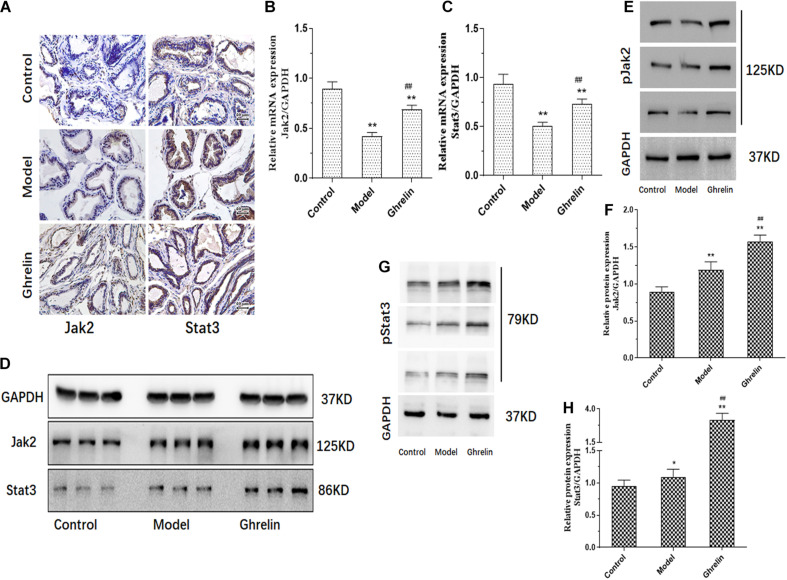
Ghrelin affects BPH development through Jak2/Stat3 signaling pathway *in vivo*. Mice after castration treated with Ghrelin (0.15 mg/kg body weight, i.p., daily for 1 week, *n* = 5) showed increased Jak2/Stat3 analyzed by immunohistochemistry **(A)**. Compared with control group (Normal mice with placebo) and Testosterone propionate (TP) (7.5 mg/kg body weight, s.c., daily for 2 weeks, *n* = 5) treated group, not only Jak2/Stat3 mRNA detected by qPCR **(B,C)** but also protein expression including pJak2/pStat3 measured by western blotting **(D–H)** was significantly increased in Ghrelin treated group. *p < 0.05, **p < 0.01 compared with Control (*n* = 5, Normal mice with placebo). ##*p* < 0.01 compared with Model (Mice treated with Testosterone propionate, 7.5 mg/kg body weight, s.c., daily for 2 weeks, *n* = 5). The samples for detection in panel **D** are the same as that in Figure 5D and GAPDH blots was the same.

Ghrelin induced mRNA and protein expression of Jak2 and Stat3 was measured in our study. Both Jak2 and Stat3 protein expression tend to be increased in prostate of mice treated with Ghrelin analyzed by immunohistochemical assay (Figure 6A). Compared with model group, both Jak2 and Stat3 mRNA expression was significantly up-regulated in prostate of mice treated with Ghrelin detected by qPCR (*p* < 0.01) (Figures 6B,C) which was lower than control group. Results of western blotting further confirmed that protein expression of Jak2/pJak2/Stat3/pStat3 was also increased in Ghrelin treated animals (Figures 6D–H). Jak2 and Stat3 tend to be activated in prostate of mice treated with Ghrelin, even though TP induced BPH mice showed much more positive expression of Jak2/Stat3 than control group.

## Discussion

Gut microbiota is related to the host health and disease closely. Imbalance of gut microbiota may affect the metabolic disorders as obesity and diabetes (Miele et al., 2015; Jing et al., 2016; Munoz-Garach et al., 2016; Xu P. et al., 2017). Microbiota can extract energy from dietary material which cannot be digested to regulate metabolism (Flint et al., 2008). Alteration of microbiota ratio generally affects the normal food digestion process. The gut microbiota is required in shaping the biochemical profile of the diet (Rowland et al., 2018) and affects the development of excessive body weight (Isolauri, 2017). Firmicutes phylum and Bacteroidetes phylum are the two major phyla of mammals’ gut bacteria. Firmicutes is reported to be involved in energy resorption and potentially related to the development of diabetes and obesity (Ley et al., 2006; Miele et al., 2015). Bacteroidetes species produce succinic acid, acetic acid, and in some cases propionic acid and plays an important role in protein metabolism. There is abundant evidence confirm that higher of Firmicutes/Bacteroidetes ratio of gut microbiota was found in obese patients (De Bandt et al., 2011; Balfego et al., 2016; Louis et al., 2016) and HFD fed animals (Huazano-Garcia et al., 2017; Wang H. et al., 2018; Cheng et al., 2019). Altered Firmicutes/Bacteroidetes ratio of gut microbiota in animals promotes increased ghrelin secretion correlated with MetS (Perry et al., 2016). In the present study, we investigated the change of Firmicutes/Bacteroidetes ratio and preliminarily explored the relation of Ghrelin and Gut microbiota in HFD induced mice. Our results indicated that BPH mice have higher Firmicutes/Bacteroidetes ratio and level of Ghrelin were significantly increased in serum and prostate tissue of BHP mice.

Induced by multiple factors, BPH is sensitive to the overall endocrine microenvironment. Despite of the 5α-Reductase inhibitors, few new medications was found in the market and very few targets of BPH have been confirmed at present, although some markers as oxytocin (Xu H. et al., 2017; Li et al., 2018) IL-17 and ANGPT2 (Arivazhagan et al., 2017) involved in BPH development are in research recently. Novel targets of BPH for treatment up to date are still required.

Benign prostatic hyperplasia refers to histological diagnosis of male lower urinary tract symptoms (LUTS) as benign prostate enlargement (BPE) and benign prostatic obstruction (BPO). More and more studies indicate that MetS and inflammation are prominent elements in men having LUTS related to BPH development (De Nunzio et al., 2012; Ngai et al., 2017). MetS comprises a cluster of risk factors as central obesity, insulin resistance and hyperlipidemia. BPH mice induced by TP and fed with HFD tend to have Hyperglycemia. Increasing evidence has supported obesity as a risk factor for BPH. BMI of BPH patients in our study tend to be higher than normal groups, and most of BPH patients are overweight. Obesity induces a few mechanisms as increased inflammation process, intra-abdominal pressure and sympathetic nervous activity as well as altered endocrine status and oxidative stress, all of which are favorable involved in the development of BPH (Parikesit et al., 2016). Increasing epidemiologic evidence suggests that hyperglycemia and insulin resistance included in diabetes associated with increased sympathetic tone, induction of systemic inflammation and oxidative stress, alterations in sex steroid hormone expression and stimulation of prostate growth by insulin, significantly increase the risks of BPH/LUTS (Breyer and Sarma, 2014). Shih et al. (2018) reported that a HFD and hyperlipidemia are associated with an increased risk of BPH.

Ghrelin is a gastrointestinal hormone, which is originally isolated from the stomach and acts through the growth hormone, which has been identified in tissues as prostate (Malendowicz et al., 2009; Lu et al., 2012). MetS is believed being correlated with Ghrelin mostly. Ghrelin affects food intake and energy metabolism and regulates glucose and insulin metabolism (Jabbari et al., 2018). Ghrelin levels associate with high insulin concentration and insulin resistance (Barazzoni et al., 2016; Jabbari et al., 2018). As a stomach-derived orexigenic peptide, Ghrelin transmits starvation signals to the hypothalamus via the afferent vagus nerve (Naznin et al., 2015). Adipocytokines were significantly higher, whereas Ghrelin declined in HFD-induced rats (Adedeji et al., 2018), however in another study by Esener et al. (2017), serum Ghrelin level was increased in HFD animals. Peripheral and central Ghrelin resistance was reported being caused by diet-induced obesity through promoting inflammation (Naznin et al., 2015). The Ghrelin-system is a complex family composed of several peptides, including native-Ghrelin and its In1-ghrelin splicing variant, and receptors (GHSR 1a/b), which have been reported being dysregulated in various tumors, including prostate cancer. Different types of Ghrelin tend to play different roles in prostate. Ghrelin exerts different effects on cell proliferation in prostate carcinoma cell lines. Ghrelin, in addition to des-acyl Ghrelin inhibited DU-145 cell proliferation as well as displayed a biphasic effect in PC-3 cells (Cassoni et al., 2004). In another study of Yeh et al. (2005) ghrelin could promote proliferation in the PC3 prostate cancer cell line through activating MAPK. According to a previous study, In1-ghrelin levels (in tissue) and circulating levels (in plasma) are increased in prostate cancer to regulate key pathophysiological processes, including cell-proliferation, migration and PSA secretion (Hormaechea-Agulla et al., 2017).

In the present study, Ghrelin or/and Ghrelin receptor was first detected in the serum and tissues for BPH patients. Ghrelin in serum of BPH patients (*n* = 30) was significantly increased compared with normal ones (*n* = 10) (*p* < 0.01). Similar to results of serum Ghrelin measure, protein expression of Ghrelin and Ghrelin receptor was almost parallel increased in prostate tissues of BPH patients determined by western blotting and immunohistochemical analysis. The total ghrelin and its’ receptor, GHSR 1 was measured in serum and tissues of BPH mice and patients. The role of other of Ghrelin family as In1-ghrelin splicing variant in benign prostatic hyperplasia would be explored in our further study. It is generally thought that inflammation and sex hormones influence the processes of which both stromal and epithelial cells of the prostate in the transitional zone proliferate, BPH occurs (Chughtai et al., 2016). Therefore, human prostate epithelial cell line (RWPE-1) and human prostate stromal cell line (WPMY-1) was chosen in our followed study to explore the role of Ghrelin in BPH induction *in vitro*. As reported in a previous study, Ghrelin may have the ability to sustain β-cell viability and proliferation to regulate pancreatic development and physiology (Napolitano et al., 2018). Hepatocyte proliferation is stimulated by Ghrelin via regulating cell cycle. Our results suggested that Ghrelin promotes cell proliferation of RWPE-1 as well as WPMY-1 determined by cell viability and cell 3D culture. We also investigated the effect of Ghrelin on cell apoptosis and the data indicated that Ghrelin significantly inhibited apoptosis of both RWPE-1 and WPMY-1. Moreover, YIL-781 attenuates prostate enlargement in HFP and TP BPH mice, WP1066 blocks ghrelin-induced cell proliferation and apoptosis inhibition, increased Bcl2 and decreased BAX expression also strengthens the evidence that Ghrelin promotes cell proliferations while inhibits apoptosis in the prostate gland through Jak2/STAT3 pathway. Effect of Ghrelin on apoptosis in prostate cells may be consistent with results in previous study that Ghrelin attenuated apoptosis of human micro vascular endothelial cells induced by high glucose/high lipid (Liao et al., 2017),endothelial and vascular of cardiovascular system (Lilleness and Frishman, 2016; Wang X. et al., 2018) and interstitial cells (Ren et al., 2018). It is worth noting that Ghrelin seemed showing more effect on RWPE-1 than WPMY-1. We explored the effect of Ghrelin on BPH pathogeny in mice subsequently. There is no significant difference in prostatic index (PI) between the mice treated with TP for 2 weeks and animals treated with Ghrelin for 1 week. It was therefore suggested that Ghrelin induced BPH in mice which were equivalent to TP induction. Activation of Jak2/Stat3 pathway stimulates a wide range of pro-survival, proliferative, and pro-angiogenic genes, therefore activators of the transcription factor this pathway is cytokines and growth factors involved in the pathogenesis of BPH (Siejka et al., 2010). Activation of Jak2/Stat3 pathway play an important role in promoting growth of both rats and human benign prostatic epithelial cells (Huang et al., 2005; Siejka et al., 2010). We evaluated the relevance between the effects of Ghrelin on BPH induction *in vitro* and *in vivo* and activation Jak2/Stat3 pathway. Our results demonstrated that treatment with Ghrelin increased Jak2/Stat3 expression in BPH epithelial and stromal cells. Enlargement prostate tissue of Ghrelin treated mice showed increasing Jak2/Stat3 expression correspondingly. Our study was consistent with previous reports that Ghrelin activated Jak2/Stat3 signaling pathway in ventricular injury (Eid et al., 2018).

In our study, there are a few limitations. Ghrelin content in serum and prostate tissue of BPH patients should perform in considerable number samples and it is better to study the correlation between serum and prostate Ghrelin levels. Furthermore, more detailed mechanism of Ghrelin action, including Ghrelin antagonist and Jak2/Stat3 pathway inhibitor application and in larger number animals should be further studied.

Gut microbiota of BPH mice was analyzed by 16s rRNA sequencing and results indicated BPH mice have the highest ratio of Firmicutes and Bacteroidetes. Ghrelin level in serum and prostate tissue was markedly increased in BPH mice. Multiple hormones contribute to the process of BPH progress. In the present study, Ghrelin and its receptor content in the serum and prostate tissue of BPH patients was measured, the effect of Ghrelin on BPH development *in vitro* and *in vivo*, and preliminary mechanism of Ghrelin action was investigated. Elevated Ghrelin and its receptor content in the serum and prostate tissue of BPH patients were observed. Ghrelin promotes cell proliferation while inhibits cell apoptosis of both prostate epithelial cells (RWPE-1) and stromal cells (WPMY-1), which confirmed by Ghrelin induced increasing expression of Bcl2 as well as decreasing BAX expression. *In vivo* study, body weight tends to have more effects on BPH mice induced by HFD, and the effect of Ghrelin on enlargement of the prostate was almost equivalent to that of TP. Furthermore, the effect of Ghrelin on prostate enlargement of mice for 1 week is similar to TP for 2 weeks. It was observed not only in BPH cells study but also in mice BPH induction that Ghrelin could up-regulate Jak2/Stat3 expression, which suggested Ghrelin may play an important role in activation of Jak2/Stat3 pathway in BPH development.

In conclusion, our results suggested that gut microbiota may associate Ghrelin which plays an important role in activation of Jak2/Stat3 in BPH development. Gut microbiota and Ghrelin might be pathogenic factors for the BPH and could be used as a target for mediation which remains further study in more samples to obtain confirmed evidence.

## Data Availability Statement

The original contributions presented in the study are included in the article/supplementary material, further inquiries can be directed to the corresponding author/s.

## Ethics Statement

The studies involving human participants were reviewed and approved by Medical Ethics Committee of Shanghai Jiao Tong University of Medicine. The patients/participants provided their written informed consent to participate in this study. The animal study was reviewed and approved by Shanghai Jiao Tong University Medical Animal Ethics Committees.

## Author Contributions

All authors listed have made a substantial, direct and intellectual contribution to the work, and approved it for publication.

## Conflict of Interest

The authors declare that the research was conducted in the absence of any commercial or financial relationships that could be construed as a potential conflict of interest.
